# Urbanization, Life-Course Migration, and Late-Life Cognitive Health Within the Mexican Context

**DOI:** 10.1093/geroni/igaf122.1869

**Published:** 2025-12-31

**Authors:** Joseph Saenz, Shane Burns

**Affiliations:** Arizona State University, Phoenix, Arizona, United States; University of Michigan, Ann Arbor, Michigan, United States

## Abstract

Urban-dwelling is linked with better cognition across diverse settings. Whether urban advantages can be attributed to urban-dwelling (e.g., health promoting resource access) or health-related migrant selection from rural-to-urban areas is unclear. Using data from the 2003–2012 Mexican Health and Aging Study, we analyzed differences in cognition levels, and cognitive decline, among individuals who self-reported growing up in rural areas, distinguishing between those who stayed in their childhood communities (“stayers”) versus “movers” and objective late-life community size (rural/urban). We accounted for demographics and early-life health/socioeconomic position. Results indicated community urbanization may benefit cognition levels, as stayers who lived in communities that urbanized throughout their lives had better cognition than stayers whose communities remained rural. However, rural-to-urban movers had better cognition levels than rural-to-urban stayers, but this was only observed for men. Our findings suggest that cognitive advantages of urban-dwelling are due to a combination of urban-dwelling itself and healthy migration.

